# Polypropylene/Lignin/POSS Nanocomposites: Thermal and Wettability Properties, Application in Water Remediation

**DOI:** 10.3390/ma14143950

**Published:** 2021-07-15

**Authors:** Abeer Alassod, Syed Rashedul Islam, Mina Shahriari Khalaji, Rogers Tusiime, Wanzhen Huang, Guangbiao Xu

**Affiliations:** 1 Key Laboratory of Textile Science and Technology of Ministry of Education, College of Textiles, Donghua University, Shanghai 201620, China; 416006@mail.dhu.edu.cn (A.A.); sri060791@gmail.com (S.R.I.); 1179127@mail.dhu.edu.cn (W.H.); 2 Chemical Engineering and Biotechnology, Donghua University, Shanghai 201620, China; 416015@mail.dhu.edu.cn; 3 College of Materials Science and Engineering, Donghua University, Shanghai 201620, China; 317001@mail.dhu.edu.cn

**Keywords:** lignin, polypropylene, sponges, hydrophobic, sorption, thermal stability

## Abstract

Compositing is an interesting strategy that has always been employed to introduce or enhance desired functionalities in material systems. In this paper, sponges containing polypropylene, lignin, and octavinyl-polyhedral oligomeric silsesquioxane (OV-POSS) were successfully prepared via an easy and elegant strategy called thermally induced phase separation (TIPS). To fully explore the behaviour of different components of prepared sponges, properties were characterized by a thermogravimetric analyser (TGA), differential scanning calorimetry (DSC), Fourier transform infrared measurement (FTIR), and scanning electron microscopy (SEM). Furthermore, wettability properties toward an organic liquid and oil were investigated. The FTIR analysis confirmed the chemical modification of the components. TGA and DSC measurements revealed thermal stability was much better with an increase in OV-POSS content. OV-POSS modified sponges exhibited ultra-hydrophobicity and high oleophilicity with water contact angles of more than 125°. The SEM revealed that POSS molecules acted as a support for reduced surface roughness. Moreover, OV-POSS-based blend sponges showed higher sorption capacities compared with other blend sponges without OV-POSS. The new blend sponges demonstrated a potential for use as sorbent engineering materials in water remediation.

## 1. Introduction

Industrial wastewater and oil spillage are considered as the main source of water pollution, which threatens human life and the marine system [1,2,3,4,5]. Moreover, water pollution creates a great loss of energy resources if not properly treated [6]. Various methods have been widely adopted to remedy water pollution, including application of booms, in situ burning, skimmers, biological treatment, microorganisms, dispersants [7,8,9,10].

However, these methods had many disadvantages, such as that their application was limited, high-cost, poorly efficient, and had adverse effects on the environment. In this case, removing pollutants by low-cost and highly absorbent materials without generating by products that may cause further concern was the main target of material scientists for decades [11]. Different materials have been developed to face the great challenge of removing oil from water surfaces, such as a sponge [12], foam [13], carbon nanofiber aerogel [14], and carbon nanotubes [15]. These materials have useful common features such as being porous, hydrophobic, oleophilic, and making recovery possible by a simple squeeze procedure. However, most of the materials still had the significant problem of high cost in chemicals.

A low-cost and efficient strategy to produce polymer-based monolith, the thermally induced phase separation (TIPS) method has been used as a typical facile process to fabricate sponges. TIPS method is based on dissolving a polymer in a solvent at an appropriate temperature, followed by cooling of the polymer solution. The phase separation happens in the cooling cycle. TIPS is a simple operation, energy-saving, and adaptable process [16,17].

Recently, we have reported a novel approach to fabricate low-cost and eco-friendly biomass-based porous materials using renewable lignin and polypropylene (PP) via TIPS used in water remediation [18].

PP is a commonly used plastic in different industrial applications, especially in wastewater treatment as a sorbent, mainly due to the low cost, high oil sorption capacity, and relatively high efficiency [19,20,21,22]. Nevertheless, its nonbiodegradability and non-renewability after use are major disadvantages, which limit its further application [22]. The previous disadvantages were important reason to search for a renewable alternative to PP was motivated by the interest in reducing the environmental footprint [23]. Among the variety of biomass materials, lignin is recommended for sustainable development. It opens new perspectives for products because it is renewable, biodegradable, biocompatible, cheap and widely available [24,25].

Furthermore, lignin is a natural organic polymer produced on a large scale from the paper and cellulose industries. At present, despite the various proposals for using lignin, only a small part (about 2%) of the 500 million tons produced annually is effectively utilized. Most of the lignin is burned or discharged into rivers, which threatens the environment [23,24]. Lignin is known as the most abundant aromatic polymer in nature, with a complex and amorphous polyphenolic molecule composed of many functional groups, enhancing its potential for usage in several material applications and production of high-added-value products [26,27].

One of the limitations of fabrication lignin and polypropylene (PP) via TIPS method was increasing water wettability with increasing lignin content in the matrix of the sponge along with a slight decrease in thermal stability with an increased lignin content of more than 10 wt%. To solve the above drawbacks by increasing lignin amount in blend, we mixed it OV-POSS with different weight ratios to fabricate the final target product.

In the past decades, the unique properties of OV-POSS received great attention for widespread application. OV-POSS can enhance fire retardation, has good mechanical properties and wettability. Where their particles form a rigid inorganic silica cage structure responsible for enhancing thermal stability [28]. Moreover, probably all composite modified with POSS could enhance hydrophobicity, which could be used efficiently in oil-water separation [29,30]. This unique structure of POSS, based on the rigid framework, made it a potential nanofiller for many applications due to enhanced mechanical and rheological properties [31,32,33,34,35].

Furthermore, OV-POSS contains a common nontoxic compatibilizer with the smallest, 1–3 nm, molecular silica [36,37] that can be effectively incorporated into polymers, such as polypropylene, polyethylene terephthalate, and polyester, using copolymerization, grafting, or simply traditional blend methods [38,39,40]. There is a very limited number of published studies on OV-POSS used as an agent with polypropylene and lignin. Seydibeyoğlu et al. synthesized reinforced composite for industrial applications using polypropylene and lignin with and without coupling agents (FUSE and POSS) via twin-screw extruder to investigate the morphology and thermal properties. The authors stated that the thermal stability and morphology of the composite were enhanced by introducing POSS [41].

Herein, we propose a facile strategy of fabricating an environmentally friendly sponge that can be used to remove and recover, via thermally induced phase separation (TIPS), oil and organic liquid from water surfaces, using the three constituents—PP, lignin, and OV-POSS—. We then investigated the effect of OV-POSS nanoparticles with different levels (1 and 3 wt%), which were also used as compatibilizers, on the wettability and thermal properties of the blends.

## 2. Materials and Methods

### 2.1. Materials

**Figure 1:**
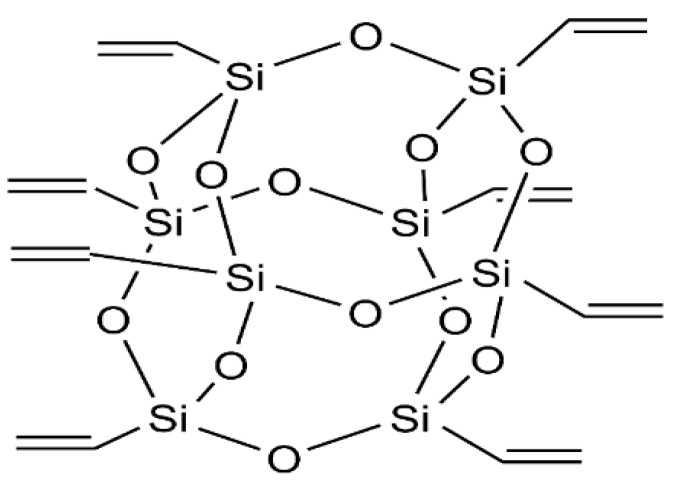
Structure of OV-POSS.

Polypropylene (PP) pellets, octavinyl-polyhedral oligomeric silsesquio ane (OV-POSS, Figure 1), lignin (L), acetone, decalin, 1-butanol, dichloromethane, and hexane were purchased from Aladdin (Shanghai, China) and used without further purification. Dichloromethane and hexane were used as organic solvents. Engine oil and soybean oil were bought at a local market (Shanghai, China) t. All reagents were used without further purification. Typical properties of the materials are reported in Table 1.

### 2.2. Fabrication of Blend Sponge

**Figure 2:**
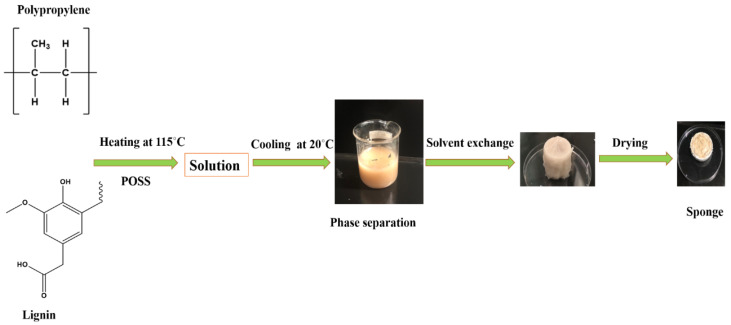
General procedure for fabrication of a blend sponge.

To fabricate the target sponges, the typical process was done as follows: The solution of PP was prepared by completely dissolving 2.8 g of PP pellets in a mixed solvent containing decalin (16 mL) and 1-butanol (24 mL), heating at a temperature of 115 °C until the polymer was completely dissolved to form a homogenous solution. Afterward, lignin and OV-POSS were added to the solution and gentle stirring followed for 1 h to obtain a uniform mixture. The solution was then cooled at room temperature for 2 h. It should be noticed that the phase separation finished in a short time. During the cooling stage, phase separation took place, forming a sponge. After that, the obtained sponge was immersed into acetone to remove the embedded solvent and subsequently dried under vacuum. The general procedure for the fabrication of the target sponge is illustrated in Figure 2. The contents of all the composites are listed in Table 2. The added levels of dried lignin and POSS were set of the total weight of PP pellets. The dried lignin was added in two different amounts, 10 and 20 wt% (total mass percentages) of PP, and the corresponding composites were labelled PP10L and PP20L, respectively. The dried OV-POSS was also added in two different amount, 0.1 and 0.3 wt%. OV-POSS was abbreviated to POSS.

### 2.3. Characterization

#### 2.3.1. Gel Permeation Chromatography Analysis of Polypropylene

The molecular weight of PP was determined by polymer laboratory Gel permeation chromatography (PLGPC-220, Shropshire, UK) at 150 °C, using 1,2,4-trichlorobenzene as a solvent, and the calibration was made with polystyrene standards.

#### 2.3.2. H NMR Spectroscopy

H NMR analysis was carried out to determine the chemical structure of the lignin, using a Bruker Avance 600 MHz spectrometer (Malaga, Spain) at a frequency of 600 MHz at 25 °C. The lignin samples were dissolved with DMSO to enhance their solubility.

#### 2.3.3. Fourier Transform Infrared (FTIR) Measurement

The infrared spectra were obtained using an FTIR spectrometer (Nicolet 6700, Thermo Fisher, Freehold, NJ, USA). The spectra were recorded at the wavenumber range of 400–4000 cm^−^^1^. The spectra were generated in the range of 400–5000 cm^−1^ using attenuated total reflection (ATR) technique. The test was used in 64 scans with a 8 cm^−1^ resolution.

#### 2.3.4. Thermogravimetric Analysis

The thermal stability of PP, lignin, POSS, and obtained polypropylene/lignin blend monoliths were investigated using a thermogravimetric analyser (TGA 4000, PerkinElmer, Freehold, NJ, USA). Sample masses of ~10 mg were heated at 30–700 °C at a heating rate of 10 °C/min under nitrogen flow (20 mL/min).

#### 2.3.5. Differential Scanning Calorimetry (DSC) Analysis

DSC analyses were used to evaluate the effect of added lignin on the polypropylene thermal behaviour of the blend monoliths. The measurements were carried out using differential scanning calorimetry (DSC 4000, PerkinElmer, Freehold, NJ, USA). Samples of ~3–5 mg in an aluminium container were carried out by heating/cooling at a rate of 10 °C/min under nitrogen atmosphere within a temperature range of 30–200 °C.

#### 2.3.6. Scanning Electron Microscopy (SEM) Characterization

The surface morphology of polypropylene/lignin sponges and their modifications with POSS were characterized using scanning electron microscopy (FLEX SEM1000, Hitachi, Chuocho, Kagoshima, Japan). For SEM, samples were cut, fixed on double tape, and then plated with a thin film of gold before measurement.

#### 2.3.7. Contact Angle Determination

The contact angles and surface free energies were measured by an optical contact angle meter (OCA15EC, DataPhysics, Filderstadt, Germany). In the testing process, double-sided tape attached the fibres to a glass slide, paved in the form of a plane [42]. Surface energies of the three samples were calculated based on the Owens–Wendt–Rabel–Kaelble (OWRK) method using three different liquids: water, ethylene glycol, and ethanol [21,43,44].

#### 2.3.8. Measurements of Oil Sorption Capacity and Reusability

The sorption capacity Q (%) was calculated from the mass gain after the obtained samples were weighed and placed in a beaker containing a 5 mm layer of liquid (oil—organic solvents) floating on an 80 mm layer of water for a duration of 30 min to reach saturation. After that, the saturated materials were weighed again; the sorption capacity was calculated by Equation (1) [45], where Wi and Wt are the weights before and after absorption, respectively.

(1) $$Q=\frac{\mathrm{Wt}-\;\mathrm{Wi}\;}{\mathrm{Wi}}\times 100$$

The reusability of sorbent was estimated by repeated sorption and squeezing processes. The sorbent material was soaked into liquid, and the absorbed liquid within the sample was recovered by manual squeezing. The process was repeated for several cycles [46].

## 3. Results and Discussion

### 3.1. H NMR Spectroscopy for Lignin

**Figure 3:**
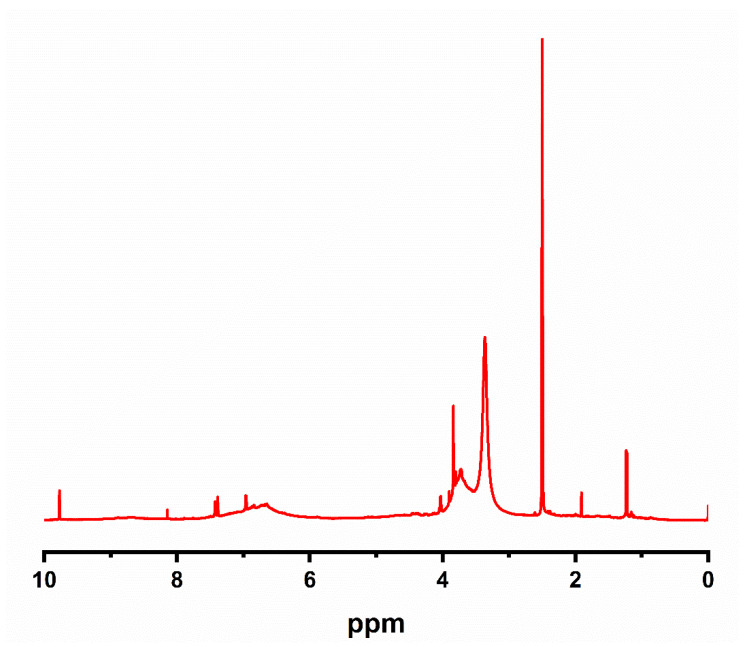
H NMR spectrometry of the sample of lignin.

The chemical structure of lignin was assessed by H NMR (Figure 3). The obtained spectra showed that the sample of lignin had signals in the range of 2.5–3.5 ppm, which was assigned to DMSO (solvent). It also displayed signals in the range of 4.0–3.5 ppm, assigned to protons in methoxyl groups. Furthermore, lignin had the most important of all signals in the range between 6 and 8 ppm, which could be attributed to aromatic protons. The signals in ranges of 7.4–7.5, 7.3–6.8, and 6–6.8 ppm were attributed to p-hydroxyphenyl (H), guaicyl (G), and syringyl (S) units, respectively [47,48].

### 3.2. FTIR Analysis

**Figure 4:**
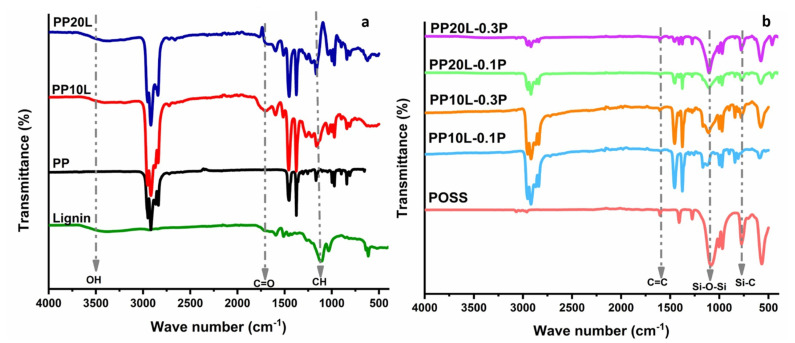
FTIR spectra of polypropylene, lignin, and polypropylene/lignin blend sponges (**a**) without POSS and (**b**) with POSS.

The Fourier transform infrared spectra (FTIR) method was used to describe the characteristics of functional groups present in the structure of the blend sponge with and without POSS (Figure 4). Polypropylene showed peaks at 2949, 2916, 2866, 2837, and 1375 cm^−1^ that were attributed to the C–H stretching. The peaks at 1375, 1358 and 1330 cm^−1^ represent the syringyl group, the –CH_3_ bonding, and the C–O stretch, respectively [18]. The spectra of all the blends had these peaks, as can be seen in Figure 4a.

Further, it was observed that the lignin peak at 3500 cm^−1^ corresponded to hydroxyl groups. This vibration of hydroxyl groups was visible also in PP10L and PP20L. On the other hand, with the addition of lignin to PP, a peak of aromatic skeletal vibrations was found at 1510 cm^−1^, which was more intense at higher lignin content. Anther essential structure in lignin was a weak shoulder at 1710 cm^−1^, which is associated with conjugated carbonyl stretching [49].

The FT-IR spectra of blend sponges with POSS are shown in Figure 4b. Strong peaks at 1060 and 1003 cm^−1^ were observed, characteristic of the Si–O–Si stretching. The peak at 1603 cm^−1^ was associated with C=C stretching vibration of the vinyl group, besides peaks around 1410 cm^−1^ assigned to C=C stretching [50]. The peak at 779 cm^−1^ was related to Si–C stretching vibration [51]. The peaks at 1595 cm^−1^ (aromatic C=C stretching), 1513 cm^−1^ (aromatic skeletal vibration rings), and 1455 cm^−1^ (C–H deformation) were visible in all the composites [16].

It was observed the above peaks, representing bonds in POSS, appeared in all blend polymers after modification with POSS. The modification of the blended sponge with POSS is based on the interaction between the POSS molecule and the hydroxyl groups in lignin, which was proven by the disappearance of the peaks of hydroxyl groups in the range of 3400–3600 cm^−1^. This indicated that lignin and POSS were successfully incorporated into the composites [52,53].

### 3.3. Thermal Gravity Analysis

**Figure 5:**
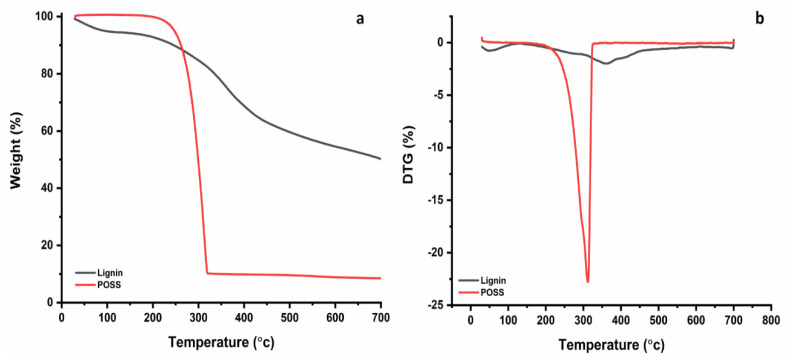
(**a**) Thermal Gravity(TG) curves of lignin and POSS, (**b**) Differential thermogravimetry (DTG) curves of lignin and POSS.

**Figure 6:**
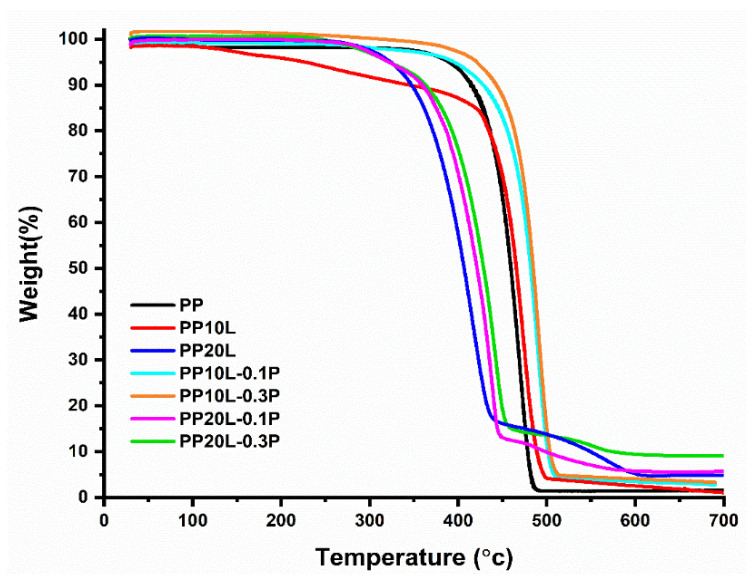
Thermal Gravity (TG) curves of polypropylene, polypropylene/lignin blend sponges, and their forms modified with POSS.

**Figure 7:**
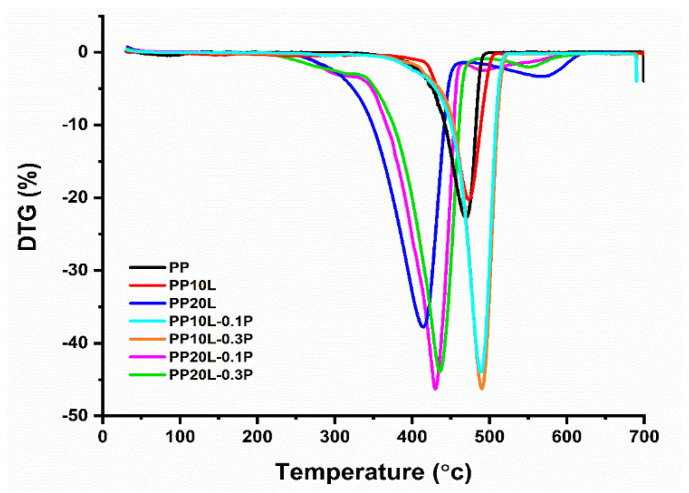
Differential thermogravimetry (DTG) curves of polypropylene, polypropylene/lignin blend sponges, and their modified forms with POSS.

Thermal gravity analysis (TGA) is a widely used tool to evaluate the thermal degradation behaviour of tested samples. The mass loss of the tested sponges was shown as a function of temperature on thermogravimetric (TG) and derivative of thermogravimetric (DTG) curves (Figure 5, Figure 6 and Figure 7). The corresponding data, such as the temperature at 5% mass loss (T5%), at 10% mass loss (T10%), and at 50% mass loss (T50%), the initial decomposition temperature (Tonset), the peak of DTG, and charred residue at 600 °C are listed in Table 3. Figure 5a, b displays the TG and DTG curves of lignin and POSS. The thermal degradation of lignin was divided into three distinct regions (Figure 5). In the first one, degradation occurred between 30 and 100 °C, where mainly the gradual evaporation of moisture can explain the weight loss. In the second region, the heavy loss of lignin degradation process occurred between 182 and 500 °C, and the DTG peak for lignin occurred at 360 °C. The third region ranged from 510 to 700 °C. According to the TG curve, the onset degradation temperature of lignin was at 225 °C, and the charred yield was found at 54.56%. Lignin has high thermal stability, which is attributed to the presence of a complex phenyl propanoid unit [54].

The thermal stability of POSS is shown in Figure 5. It was observed that POSS followed one single step with degradation starting at 263.50 °C with DTG at 311.12 °C and the char yield at 600 °C was around 8.88% [55].

Moreover, it was observed that PP exhibited a one-step degradation process in the temperature range of 30 to 700 °C (Figure 6). Meanwhile, a modified blend sponge with lignin content 10%, with and without POSS, showed similar degradation behaviour in the same temperature range. In contrast, the blend sponges with a lignin content of 20% had two strong peaks (the corresponding DTG curves are in Figure 7 and all parameters in Table 3). This suggests that the chemical modification does not change the degradation mechanism.

Polypropylene started to degrade at 468.92 °C, the degradation continued until 497.65 °C with a remarkable char residue (1.40%) at 600 °C. Moreover, this behaviour was also observed in the thermal behaviour of composites blended with 10–20 wt% lignin. However, the results showed clearly that the addition of lignin at 10% would increase the onset of the temperature of the thermal degradation more than PP itself. In comparison, the thermal test analysis showed that a higher content of lignin in the blend matrix would reduce its thermal stability, as it was apparent on the tested sample PP20L, where the onset of the temperature of the thermal degradation was lower than the one of PP [56]. At the same time, the percentage of charred residues of the blend monolith at 600 °C was of 2.63 and 3.47% higher than for PP itself. This result is explained by the aromatic chemical structure of lignin in the blends, which gives high amounts of char at the evaluated temperature [57].

The influence of POSS on the thermal behaviour of blends was also investigated. In the case of the work carried out under nitrogen atmosphere, it was notable the addition of POSS into the matrix of the sponge caused an increase in T5%, T10%, and T50% compared with a blended sponge without POSS (Table 3). Increasing POSS content in the matrix increased these values.

Generally, there was an increase in the Tonset under the nitrogen atmosphere, which w attributed to possible cross-linking between blend chains and the POSS silicone core, and a thermally stable ceramic char surface layer was formed by POSS cage and acted as a thermal barrier for materials. The amounts of POSS present in the blends also affected the thermal stability due to significant enhancement with POSS. The increases in decomposition temperatures of PP10L-0.1P and PP20L-0.1P were 455.61 and 388.22 °C, whereas in the case of PP10L-0.3P and PP20L-0.3P, they were 463.78 and 399.50 °C, respectively.

Further, all POSS used here improved the temperature of the maximum weight loss rate (Figure 6). Another essential factor that was observed in the TG analysis was the charred residue yield. With an increase in the quantity of the inorganic additive POSS in the blend sponge, the percentage of charred residues increased [58]. The increase in char residue yield with increasing POSS content was of 4.55% (PP10L-0.1P), 7.11% (PP20L-0.1P), 5.30% (PP10L-0.3P), and 10.56% (PP20L-0.3P), as listed in Table 3.

### 3.4. Differential Scanning Calorimetry (DSC)

**Figure 8:**
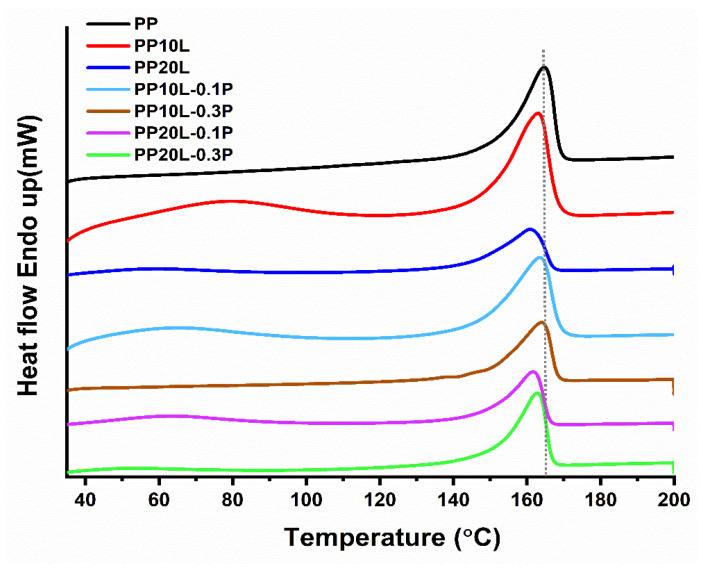
Differential Scanning Calorimetry (DSC) curves of polypropylene, polypropylene/lignin blends sponges, and theirs modified with POSS.

The DSC curves of polypropylene and its blends with and without POSS are shown in Figure 8. The relative parameters, such as melting temperature (Tm), melting enthalpy (∆Hm), and degree of crystallinity (X_C_), are summarized in Table 4. The percentage crystallinity (Xc) of PP and its blends with lignin and POSS was calculated according to the following Equation (2).

(2) $$X_{C}=(\frac{\Delta H_{m}}{W\;\Delta H_{m}^{o}})\times \;100\%$$

where W is the mass of the PP in the blends,$\;\Delta H_{m}\;$is the melting enthalpy of PP and their blends, $\Delta H_{m}^{o}$ (209 J/g) is the reference value that denotes the melting enthalpy of PP crystals [59].

It was observed that neat PP had a melting enthalpy$\;\Delta H_{m}$ of 101 J/g, whereas the blend sponge with lignin had lower melting enthalpy values; this was due to the incorporation of lignin. Furthermore, the melting temperature of the blend was lower than that of neat PP, which might be explained by the low molecular weight of lignin which acts as a plasticizer [60]. However, the addition of POSS to the mixture imparted a slight increase in relative parameters. The melting temperature of the blend sponge increased with increasing the POSS content. This demonstrated that the heterogeneous crystal nucleation of the blend can be enhanced by the silica nanoparticle content of POSS [61].

### 3.5. Morphology Observation

**Figure 9:**
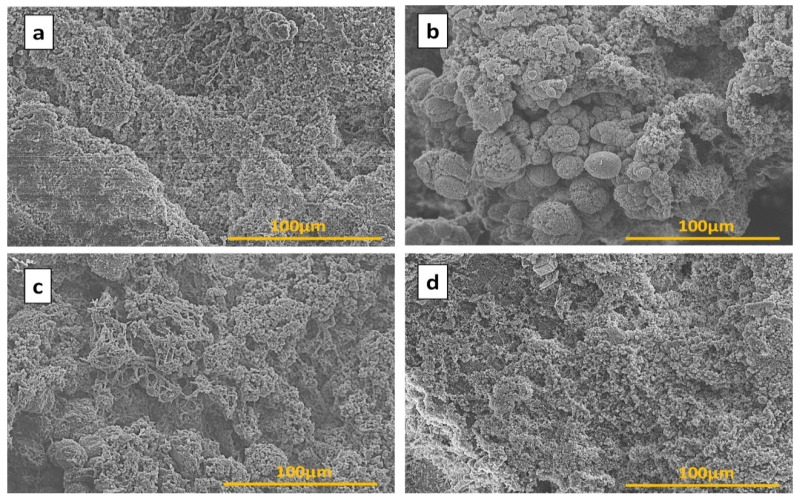
SEM images of (**a**) PP, (**b**), PP10L (**c**), PP10L-0.1P, and (**d**) PP10L-0.3P.

Surface topography is an important factor for studying the wettability of materials surfaces. Figure 9 shows the SEM images of PP, PP10L, and their forms modified with POSS. The surface of the blend sponge without POSS seemed to be rough due to lignin aggregations (Figure 9b). The tested sponge also displayed a turbid nature due to the phase separation. However, the surface of the blend sponge with POSS was rather smooth with no detected POSS particles on the surface (Figure 9c,d). With increasing POSS content in the blend sponge, its surface became smoother [62]. In this case, the nanoparticles of POSS improved the dispersed lignin particles in the sponges and formed a good surface condition [63].

### 3.6. Surface Properties

**Figure 10:**
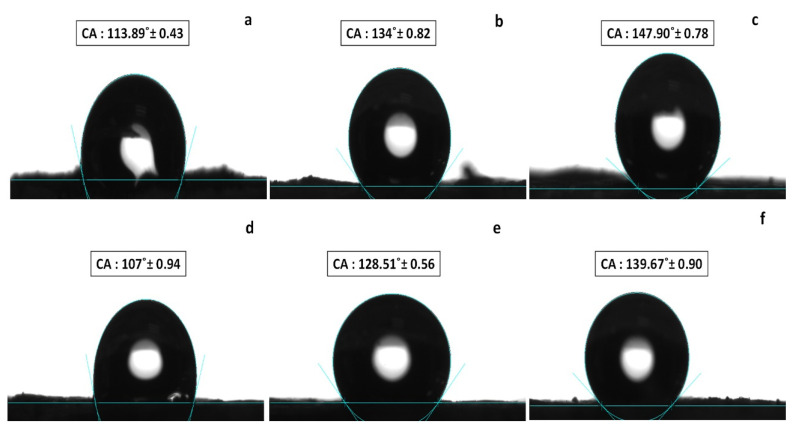
Water contact angles for (**a**) PP10L, (**b**) PP10L-0.1P, (**c**) PP10L-0.3P, (**d**) PP20L, (**e**) PP20L-0.1P, (**f**) PP20L-0.3P.

The surface wettability of the samples is another interesting factor; it was studied by measuring the static contact angles between samples and water. The measured surface free energies are summarized in Table 5. The incorporation of POSS within the blends was essential to the surface energy and surface hydrophobicity [64]. It can be deduced from Figure 10 and Table 5 that with an increase in the POSS content, the hydrophobicity of tested samples was clearly enhanced. The water contact angle of PP20L increased from 107° to 128.51° with 0.1% POSS and increased to 139.67° with 0.3% POSS, which meant more hydrophobic POSS molecules migrated to the surface of the samples as POSS content increased. Incidentally, the incorporation of POSS in PP10L and PP20L was necessary to lower the surface free energy, as already stated in Table 5. The presence of the Si–O–Si network structures in the blend matrix provided thermodynamic motion from the inside to outside and a smoother surface, resulting in reduced surface free energy.

### 3.7. Organic Solvents/Oil Sorption

**Figure 11:**
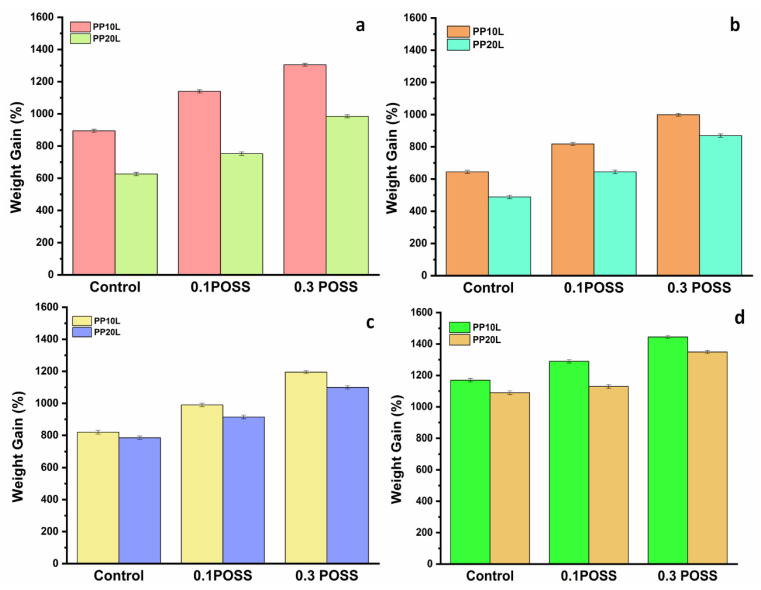
Absorption capacities of polypropylene and blend sponges modified with POSS. (**a**) Soybean oil, (**b**) engine oil, (**c**) hexane, and (**d**) dichloromethane.

The blended sponges fabricated using the TIPS method can be considered as promising sorbents for oil spillage clean-up due to their 3D interconnected macroporous structures, hydrophobicity, and oleophilicity, resulting from mixing of lignin and POSS support micro/nanoscale structures, as discussed above. For further investigation of the absorption capacities of these blends of polypropylene/lignin with and without POSS for potential application in the clean-up of polluted oil spills, more experiments were set up using oils and organic solvents. The results are demonstrated in Figure 11 and Table 6.

**Figure 12:**
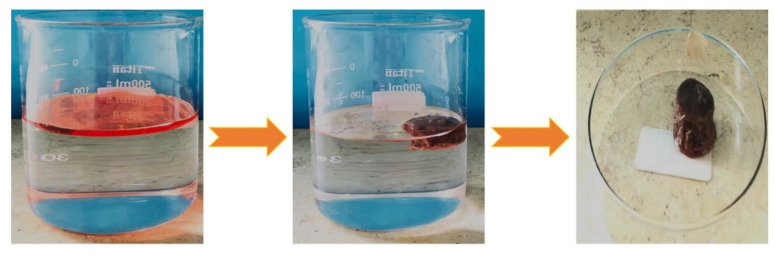
Photographs of the sorption process of soybean oil by sponge PP10L-0.3 P.

The synthesized sponges displayed good absorption capability for the various liquids and oils used. Their absorption capacity was evaluated as follows: A piece of the sponge was placed in contact with liquids dispersed on a water surface, as shown in Figure 12. The floating liquid was absorbed into the sponge via capillary forces in only a few minutes. Showing the excellent sorption capacity of the blend sponge.

**Figure 13:**
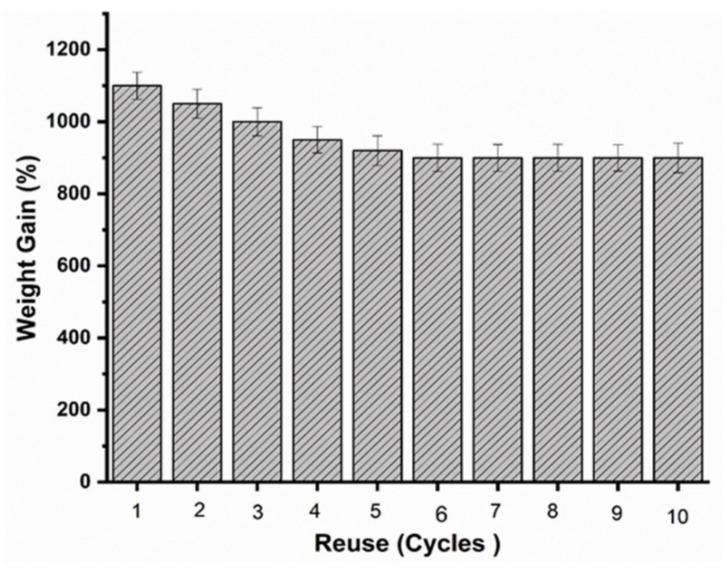
Recyclability of the PP10L-0.3 P sponge for the sorption of hexane.

Further, it was noticed that sponges modified with POSS exhibited higher sorption capacity due to the improved hydrophobicity, which ensured that water was completely rejected. It was also evident that there was a clear correlation between the volume of absorbed oil and the POSS content, which could be attributed to the increased lipophilic properties with an increase in POSS content. For example, sponges with 0.3% POSS absorbed oil more than sponges with 0.1% POSS [65]. When the sorbent sample was placed on the water surface, it floated. The absorbed oil was easily recovered by repeatedly squeezing the oil-laden sorbent without losing its excellent hydrophobicity. The absorbed solvent was collected by simple manual squeezing repeatedly, as seen in Figure 13. Furthermore, the sponge could be reused after easy washing with volatile liquid and then drying at room temperature. The sorption capacities of these blend sponges was further compared with those from previous studies (Table 7).

## 4. Conclusions

In summary, we presented a facile method of fabrication eco-friendly blend sponges from PP, lignin, and POSS using an efficient way, namely, the thermally induced phase separation (TIPS). The influence of POSS on the surface wettability and thermal properties was deeply studied. The newly prepared sponges displayed high thermal stability with POSS compared with one without POSS. A DSC analysis showed that POSS had a significant influence on melting temperatures. SEM results indicated that the addition of POSS nanoparticles could enhance the dispersion and compatibility of the lignin in matrix systems by making the surface smoother.

Meanwhile, POSS formed silicon bonds that protected the sponges from water penetration with water contact angles more than 125°, combined with improving oleophilicity, which significantly enhanced with increasing POSS content. The sorption tests for obtained sponges showed that POSS-based sponge absorbed oils and organic solvents more, and the sponge with POSS content exhibited higher sorption compared to the one without POSS. The experimental results of the sorption test also indicated that the blend sponge with an addition of 10% lignin and 0.3% POSS had the best performance. Hence, the proposed eco-friendly and inexpensive sponges with good thermal stability and wettability could be feasibly applied in water cleanup operations on an industrial scale.

## Figures and Tables

**Table 1:** Information about materials used in this work.

Material	Description	Manufacturer
Polypropylene	Melt flow index, MFI = 1.65 g/10 min Syndiotactic Mw = 358738 Mn = 75505 PD = 4.75	Bio-Chem Technology Co., Ltd., shanghai, China
Lignin (dealkaline)	Purity ≥99% Source: pulp–brown powder with particle size ~100–250 µm Mw = 2820 Mn = 1315 PD = 2.14 Water = 1.89% Ash = 0.92%	Shanghai Macklin Biochemical Co., Ltd., shanghai, China
Octavinyl-polyhedral oligomeric silsesquioxane (OV-POSS)	Purity ≥98% White solid powder with particle size in the range of 1–3 nm and dried under vacuum before use Mw = 633.04 g/mol	Zhengzhou Alfa Chemical Co., Ltd., Zhengzhou, China
Decalin	Purity ≥99% Mw = 138.25 g/mol	Bio-Chem Technology Co., Ltd., shanghai, China
1-butanol	Purity ≥98% Mw = 74.12 g/mol	Bio-Chem Technology Co., Ltd., shanghai, China
Dichloromethane	Purity ≥99% Organic solvent	Yantai Yuandong Fine Chemical Co., Ltd., Yantai, China
Hexane	Purity ≥98% Organic solvent	
Acetone	Purity ≥98% Solvent	
Engine oil	Viscosity = 234.50 mPa⋅s	Local market (Vangurd, Songjiang, Shanghai)
Soybean oil	Viscosity = 65.30 mPa⋅s	

Mw—the weight-average molecular weight; Mn—number-average molecular weight; and PD—polydispersity.

**Table 2:** Fabricated composites and their sample codes.

Sample Name	PP %	Lignin %	POSS %
PP	100	0	-
PP10L	90	10	-
PP 20 L	80	20	-
PP10L-0.1P	90	10	0.1
PP10L-0.3P	90	10	0.3
PP20L-0.1P	80	20	0.1
PP20L-0.3P	80	20	0.3

**Table 3:** Results of Thermal Gravity analysis (TGA) and Differential thermogravimetry (DTG) of polypropylene, polypropylene/lignin blends sponges and their modified forms with POSS.

Samples	T5%, °C	T10%, °C	T50%, °C	Tonset, °C	DTG1, °C	DTG2, °C	Residue, %
PP	389.90	414.99	458.91	419.61	468.92	-	1.40
PP 10L	226.83	342.26	465.23	428.49	474.23	-	2.67
PP10L-0.1p	395.14	426.83	481.63	455.61	488.69	-	4.55
PP10L-0.3p	423.82	444.41	485.63	463.87	491.75	-	5.30
PP 20L	330.18	347.55	406.24	361	414.20	568	3.47
PP 20L-0.1P	339.09	360.34	422.17	388.22	433.15	554.63	7.11
PP 20L-0.3P	343.24	365.17	429.51	399.50	439.95	493.53	10.56

**Table 4:** Differential Scanning Calorimetry (DSC) data of PP and their composites.

Samples	Tm (°C)	∆Hm (J/g)	XC (%)
PP	165.22	101	48.32
PP 10L	163.01	79	41.99
PP10L-0.1p	163.40	88	46.78
PP10L-0.3p	164.38	90	47.84
PP 20L	160.80	63.80	38.02
PP 20L-0.1P	161.40	70	41.86
PP 20L-0.3P	162.85	72	43.06

**Table 5:** The contact angles with water and the surface free energy **^a^**.

Sorbent	Contact Angle (°)		$\mathrm{Surface}\;\mathrm{Energy}\;(\mathrm{mN}\cdot m^{-1})$			
	Water	Ethylene Glycol	Ethanol	Total	Dispersion Components	Polar Components
PP	127.40 (0.73)	99 (0.89)	36 (0.91)	43.55	38.29	5.26
PP10L	113.89 (0.78)	60.45 (0.97)	11.56 (0.89)	38.14	37.84	0.30
PP20L	107 (0.94)	43 (0.78)	8.90 (0.67)	41.20	41.19	0.01
PP10L-0.1P	134 (0.82)	105 (0.89)	25 (0.77)	33.97	30.03	3.93
PP10L-0.3P	147.90 (0.78)	125 (0.88)	40 (0.75)	31.05	24.81	6.24
PP20L-0.1P	128.51 (0.56)	93 (0.78)	21 (0.67)	36	33.02	2.98
PP20L-0.3P	139.67 (0.90)	111 (0.94)	28 (0.66)	34.74	29.52	5.23

Note: Values within parentheses refer to the standard deviations for five repeats.

**Table 6:** Weight gain of sponges with tested liquid (soybean oil, motor oil, hexane, and dichloromethane).

Samples	Weight Gain %			
	Soybean Oil	Motor Oil	Hexane	Dichloromethane
PP10L	895	627	1090	1170
PP20L	645	489	785	820
PP10L-0.1P	1140	753	1130	1290
PP10L-0.3P	1305	985	1350	1445
PP20L-0.1P	818	645	915	990
PP20L-0.3P	1000	870	1100	1195

**Table 7:** Sorption of sorbents.

Sorbent	Oil Sorption		Reference
PP sponge	100% 110% 75%	pump oil soybean oil n-hexane	[12]
Lignin	Up to 70%	carotino oil	[66]
PP10L-0.1P	1140% 753% 1130% 1290%	soybean oil engine oil hexane dichloromethane	current study
PP10L-0.3P	1305% 870% 1350% 1445%	soybean oil engine oil hexane dichloromethane	

## Data Availability

Not applicable.
